# Twin Reversed Arterial Perfusion Sequence With Acardius Acormus: The Role of 3D Imaging and Pelvimetry in Obstetric Planning

**DOI:** 10.7759/cureus.92924

**Published:** 2025-09-22

**Authors:** Victoria M Estevez, Alexandria Zarilla, Murat Ibatullin, Kamil Yusupov

**Affiliations:** 1 School of Medicine, Lake Erie College of Osteopathic Medicine (LECOM), Bradenton, USA; 2 Medical Imaging, Lake Erie College of Osteopathic Medicine (LECOM), Bradenton, USA; 3 Ultrasound, Kazan State Medical University, Kazan, RUS; 4 Ultrasound, Interregional Clinical Diagnostic Center, Kazan, RUS

**Keywords:** acardius acormus, advanced imaging, monochorionic pregnancy, placental arterio-arterial anastomoses, twin reversed arterial perfusion (trap)

## Abstract

Twin Reversed Arterial Perfusion (TRAP) sequence is a rare complication of monochorionic pregnancies in which an acardiac twin is perfused retrograde via placental arterio-arterial anastomoses from a structurally normal "pump" twin. We present the case of a G2P1 (two pregnancies and one delivery at a viable gestational age) 27-year-old woman at 33 weeks of gestation referred for an ultrasound due to a suspected placental tumor. Imaging revealed a normally developing fetus and an additional malformed fetal-like structure with reversed blood flow, leading to a diagnosis of acardius acormus, a rare morphological subtype of the TRAP sequence. This case highlights the utility of advanced imaging modalities, including 3D ultrasound, Doppler studies, and MRI, in the diagnosis, monitoring, and delivery planning of complex twin pregnancies involving TRAP.

## Introduction

Acardiac twinning, seen exclusively in monochorionic pregnancies, occurs in roughly 1% of monozygotic twin gestations and about 1 in 35,000 overall pregnancies [1]. Acardiac results from an abnormal vascular connection within the placenta. This leads to the healthy (pump) twin perfusing the malformed (acardiac) twin through arterio-arterial and veno-venous anastomoses [2]. The acardiac twin then receives poorly oxygenated blood, impeding proper organogenesis and leading to a variety of structural anomalies [2].

The Twin Reversed Arterial Perfusion (TRAP) sequence develops early in the first trimester, typically before 13 weeks, due to the lower vascular resistance in smaller embryos, enabling reverse perfusion [3]. Subtypes are defined by the degree and region of morphology, with acardius acormus being among the rarest [4]. Acormus is described by the presence of a head or head-like structure attached to the placenta via a short umbilical cord, without any body or limbs [4,5]. Features include a single umbilical artery, reversed arterial perfusion, and severely malformed central nervous structures due to a lack of perfusion [6,7].

The pump twin in these pregnancies is at high risk for complications due to the stress of supplying both fetuses. Challenges include high-output cardiac failure, polyhydramnios, hydrops of the pump twin, and preterm delivery, necessitating close monitoring and tailored management [2,8,9]. If early imaging modalities are employed, these complications could be identified, and prenatal interventions, such as fetoscopic ligation or laser coagulation of the acardiac twin’s umbilical cord, could be implemented. 

Imaging modalities, including 3D ultrasound, Doppler, and MRI, play an important role in identifying vascular anomalies, fetal morphology, and assessing perinatal risks [8,10]. In this case, pelvimetry may be a useful evaluative tool, even though it is not routinely performed, given the significant anatomical distortion seen in acardius acormus. It can aid in delivery planning and help reduce obstetric complications [11,12]. The use of these techniques may help prevent complications in the birthing process, especially when used during complicated deliveries, such as in the case of conjoined twins or acardiac twins, where the possibility of surgical intervention becomes likely [13].

## Case presentation

**Figure 1:**
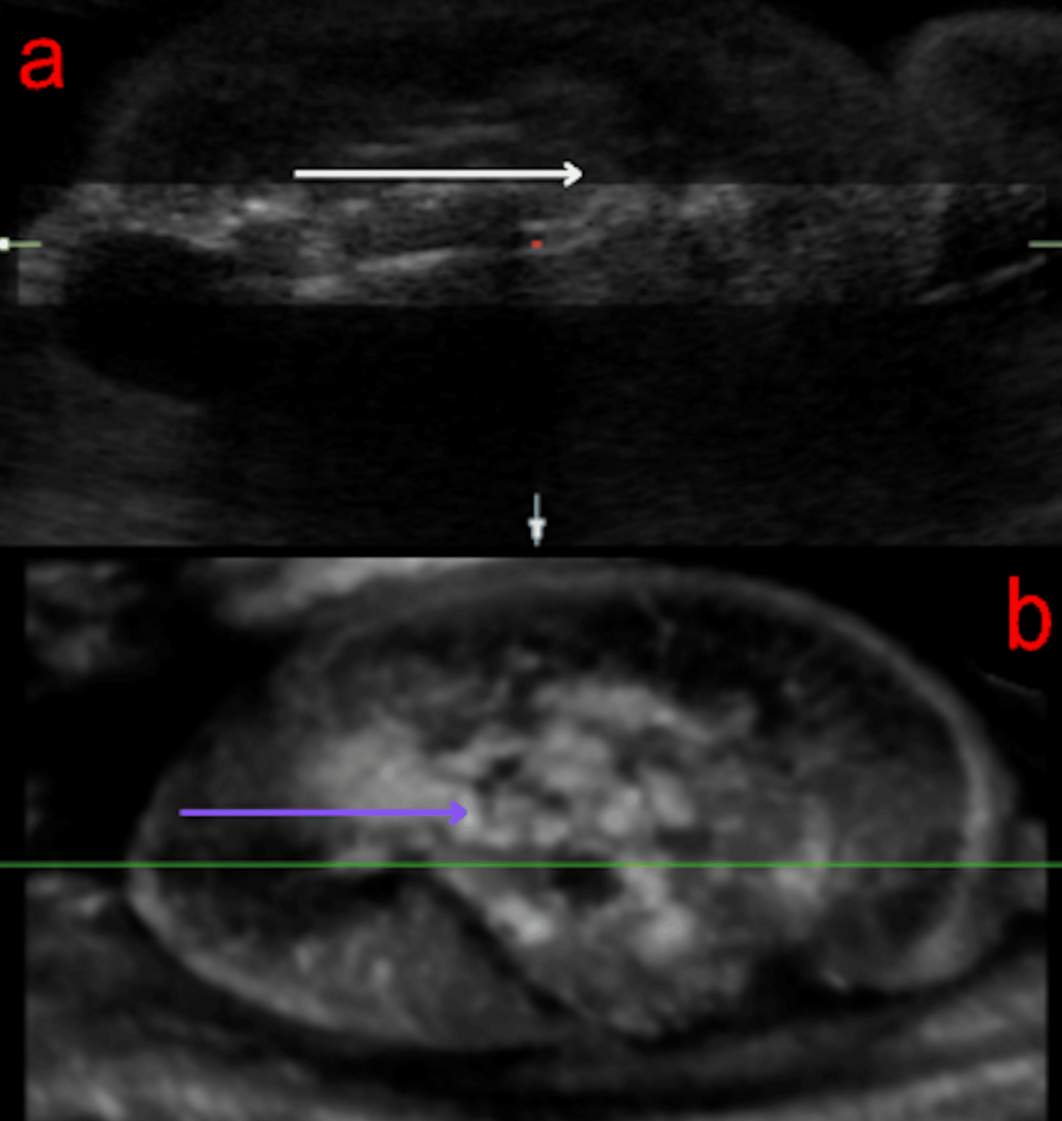
Three-dimensional multiplanar ultrasound rendering of acardius acormus. All body structures appear underdeveloped and disorganized, as indicated by the white arrow in panel (a) and the purple arrow in panel (b).

**Figure 2:**
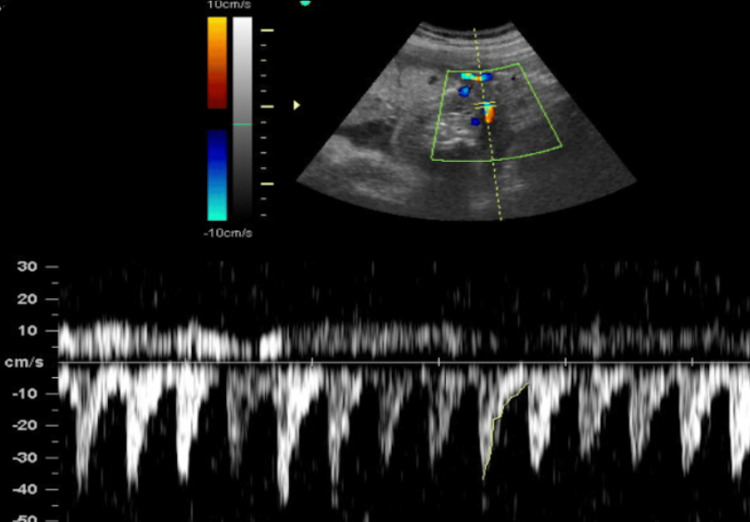
Two-dimensional umbilical Doppler. Above the center line (positive): color flow mapping; below the center line (negative): pulsed-wave Doppler.

A G2P1 (second pregnancy with one previous term delivery) 27-year-old woman, at 33 weeks of gestation, was referred for an ultrasound due to suspicion of a placental tumor. 2D and 3D multiplanar ultrasound revealed a healthy fetus measuring at 31-32 weeks and an additional mass characterized by a hyperechoic core resembling bone and a surrounding anechoic halo (Figure 1). The mass had a short umbilical cord with one artery and one vein, and Doppler analysis demonstrated reversed arterial perfusion and biphasic venous flow synchronous with the normal fetus’s heart rate (Figure 2).

**Figure 3:**
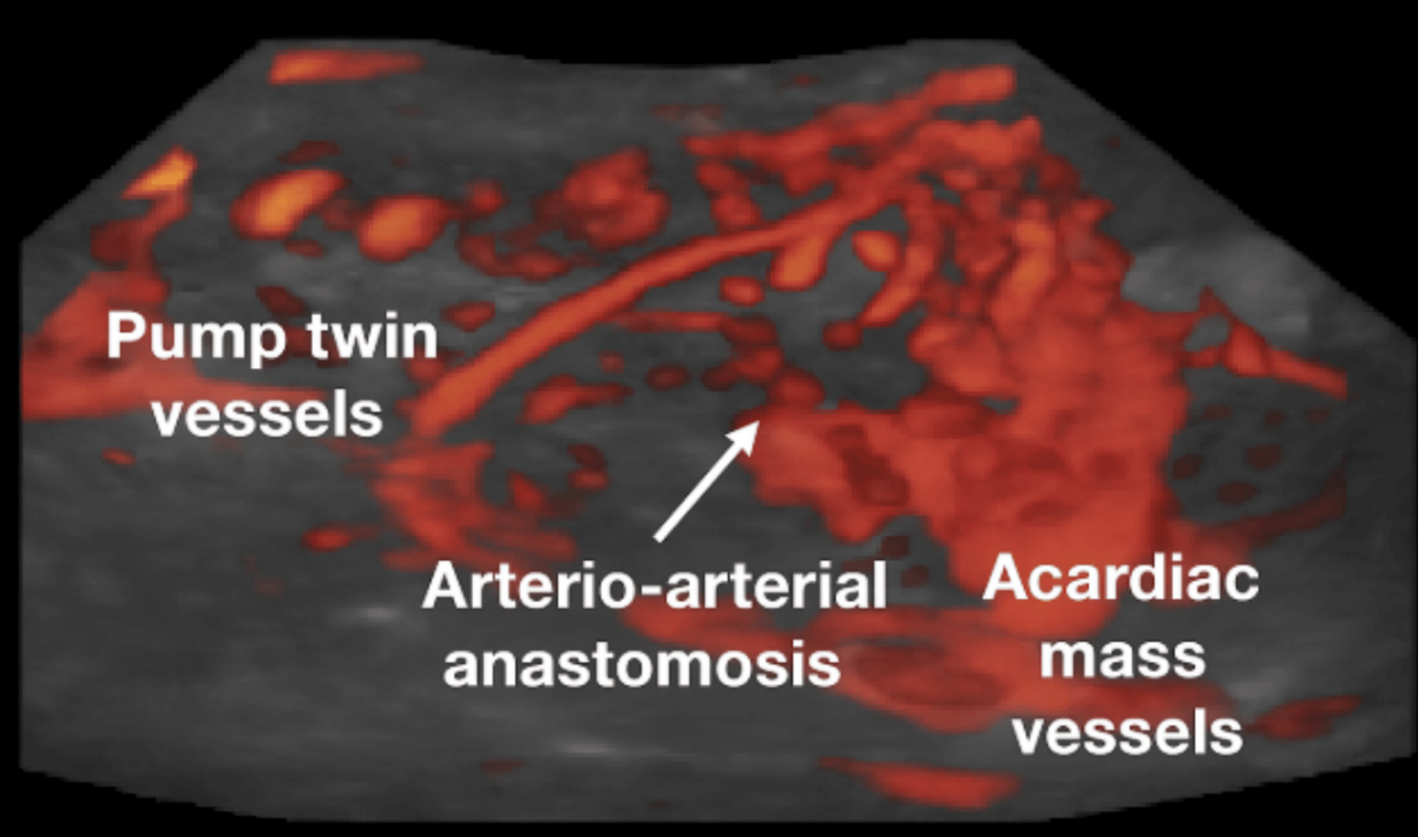
Three-dimensional (3D) power Doppler angiography with glass-body rendering.

**Figure 4:**
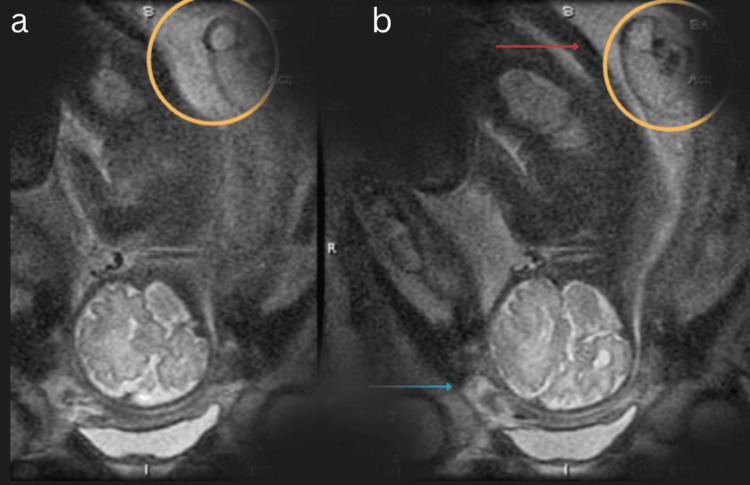
T2-weighted coronal MRI using single-shot fast spin-echo (SSFSE). Views demonstrate a head-forward fetal position, with the blue arrow in panel (b) indicating the face of the pump fetus. The orange circles in panels (a) and (b) highlight an additional inhomogeneous mass adjacent to the fetus’s lower back, indicated by the red arrow in panel (b). Note: This imaging sequence is considered safe in pregnancy, as it is rapid and does not use contrast or ionizing radiation.

Further vascular studies revealed a single feeding vessel with chaotic multidirectional flow within the mass (Figures 2-3), and MRI confirmed the diagnosis of TRAP sequence, with the malformed structure being consistent with acardius acormus (Figure 4), a rare subtype where the acardiac twin is represented only by a head with no developed body or neck. 

Figure 3 shows the vascular network within the acardiac mass in a TRAP sequence pregnancy, highlighting reversed arterial perfusion via placental arterio-arterial anastomoses from the pump twin. The bright orange-red areas are blood vessels, with the color showing where blood is moving. The gray background is the surrounding tissue, fetal and placental structures, which have been made semi-transparent so the vessels stand out.

The large, branching vessels are part of the abnormal blood supply in a TRAP sequence pregnancy. In this condition, the healthy *pump* twin sends blood through placental artery-to-artery connections into the malformed *acardiac* twin, which lacks a functional heart.

The combination of these imaging techniques was utilized to detect abnormal vascular connections (power Doppler angiography) and get a clear visualization of the malformed fetal mass and its feeding vessels without tissue layer obstruction (glass-body rendering). 

By 37-38 weeks, spontaneous labor occurred, resulting in the delivery of a healthy infant weighing 2,760 g and a second malformed fetus-like formation weighing 1,250g. The latter had an ovoid shape, skin covering with sparse hair, and a short umbilical cord attached to the head. The placenta measured 150 mm and supported two cords: one normal and one short, with two vessels.

Pathological evaluation of the acardiac mass revealed disorganized cortical architecture, near absence of neurons, extensive gliosis, focal mineralization, and agenesis of the cerebellum and brainstem. A single cryptophthalmic eye was present, with altered bone structures at the base resembling a rudimentary skull.

## Discussion

TRAP sequence, and specifically the acardius acormus subtype, poses significant diagnostic and management challenges due to its rarity and the severe anatomical abnormalities involved (Table 1). This rare variant is theorized to result from early hemodynamic imbalance in placental blood flow, allowing retrograde perfusion during the first trimester, disrupting normal embryogenesis [3,7].

**Table 1:** Classification system for acardiac twinning based on the degree of structural malformations in the acardiac fetus.

Classification	Malformation
Acardius acephalus	Absent head, well-developed trunk and limbs
Acardius amorphous	Unrecognizable fetal form
Acardius anceps	Poorly formed head, well-developed trunk and limbs
Acardius acormus	Presence of only a fetal head

In this case, advanced imaging modalities such as 3D ultrasound, Doppler, and MRI were critical not only in diagnosing the acardiac twin but also in evaluating vascular dynamics, confirming the subtype, and guiding the birth plan [5,8]. Accurate assessment of reversed perfusion and identification of key anatomical features, including a single feeding vessel and distorted cranial development, allowed for conservative management until delivery.

Pelvimetry, particularly when performed with 3D imaging or low-dose stereoradiography, serves as an important adjunct tool in high-risk pregnancies and is considered safe in pregnancy because it is fast and does not use contrast or radiation. Though not routinely recommended for all deliveries, pelvimetry becomes highly valuable in cases of TRAP sequence where fetal distortion, twin size discrepancy, or structural anomalies may influence the safest mode of delivery [3,10]. In this case, pelvimetry could assess pelvic adequacy and optimize the delivery strategy, especially given the abnormal anatomy and mass-like nature of the acardiac twin. This case emphasizes the interdisciplinary role of imaging - both fetal and maternal - in managing rare complications and improving outcomes.

## Conclusions

This case of acardius acormus highlights the essential role of advanced prenatal imaging and individualized obstetric planning in optimizing outcomes for pregnancies complicated by TRAP sequence. Although routine pelvimetry is not standard practice, its selective use in complex presentations can be critical for determining the safest mode of delivery, assessing maternal pelvic constraints, and proactively managing both maternal and fetal risk. In rare, high-risk pregnancies, the strategic integration of maternal and fetal imaging is not only beneficial but essential for delivering precise, evidence-based, and patient-centered care.
